# Physicians and Apothecaries in Puritan New England

**Published:** 1898-01

**Authors:** Herbert C. Varney

**Affiliations:** St. Paul, Minn.


### PHYSICIANS AND APOTHECARIES IN PURITAN NEW ENGLAND.

BY HERBERT C. VARNEY, ST. PAUL, MINN.

Of physicians and apothecaries in early New England it may almost be said that for the first hundred years there were none; that is, in the sense of the word as we understand it.

It must not be inferred from this, however, that our Puritan forefathers were without medical attendance; far from it, but the physicians in those days were the ministers; these were men of education and learning, nearly all of them being graduates of the University of Cambridge in England, and those of a later date of Cambridge in New England,

Many of them had been ejected from their livings for non-conformity, and some of them had taken up the practice of medicine to obtain a living. Others, after coming to this country, in order to support themselves—their pastoral salaries being insufficient—studied a few medical books, and then ministered to their neighbors in both spiritual and temporal things. It was in the hands of such men that the practice of medicine was placed for nearly the first hundred years of New England history.

The first New England physician of whom I find any record is Dr. William Gager, who came out in the spring of 1630, probably in the same fleet with Winthrop. Not being used to life in a new country, he could not endure the hardships of his new surroundings, for his death is recorded at Charlestown in September of the same year. John Dean, or, as he styled himself, "John Dean Chirurgeon" was an early physician of Ipswich, Mass. He died in 1648. His son, Philemon Dean, was one of the early physicians of his native town, renowned both for his skill in medicine and for his standing in the meeting-house. His gravestone, with its quaint Puritan epitaph, still stands in Ipswich.

> "Here lies ye body of Doctor Philemon Dean who died October ye 18th, 1716, aged 70 years.
> O Lord, by sad and awfull stroaks,
> Of man's mortality;
> O let us all be put in mind
> That we are born to dye.
> Grave saint behind that cannot find,
> Thy old love, night or morn.
> Pray look above, for there's your love
> Singing with ye first born."

The last two lines of this stanza were quite popular among Puritan epitaph makers.

Another famous physician of Ipswich was Dr. Thomas Wells, more often spoken of in the old records as Deacon

Wells. He died in 1666. By his will he left to his son "phissic bookes" valued at about nine pounds.

Some of the early physicians who came to New England found the practice of medicine, but a "meene help;" at least Giles Firmin of Boston found it to be such. He came in 1633, and is spoken of in the records as a "godly man, an apothecary of Sudbury in England." In an account of his life written by himself, he says: "Being broken from my study in the prime of my years, from eighteen to twenty-eight, and what time I could get in those years, I spent in the study and practice of physick in that wilderness." The wilderness afforded him but a very "meene" living, for in a few years we find him back in England again, and exchanging the profession of medicine for that of theology, when he became quite a noted minister. He has left one tribute to his early New England neighbors which is worthy of repetition. Before a meeting of divines held at Westminster in 1654, he said: "I have lived in a country seven years; all that time I never heard one profane oath and all that time never did I see a man drunk."

The most famous of the early physicians of Boston was Dr. John Clark, who had also settled for a time at Newburg. He was the first physician in this country to perform the operation of trepanning the skull. Dr. Clark died in 1664, aged sixty-six years. He had a son, also a doctor, who was one of the early settlers of Rhode Island.

James Minott was a physician at Concord, and from the inscription on his gravestone must have been a little of everything else.

"Here is interred the remains of James Minott, Esq., A. M., An excelling Grammarian, Enriched with the gift of Prayer and Preaching, a commanding Officer, a Physician of great Value, a great lover of peace as well as of Justice, and which was his greatest glory, a Gentleman of distinguished Virtue and Goodness, happy in a virtuous Prosperity, and living Religiously died Comfortably, September 20, 1735, aged eighty-three years."

The first women who tried to engage in the practice of medicine in Massachusetts do not seem to have met with very good success. The famous Anne Hutchinson tried her hand at medicine, but on account of her religious opinions was banished from the colony. Another, Margaret Jones, of Charlestown, was indicted and found guilty of witchcraft upon the following evidence:

1. She was found to have a malignant touch, so that whosoever she touched were taken with deafness and vomiting.

2. She practiced physic; her medicines being harmless; such as anise seed and liquors, yet they had extraordinary and violent effects.

3. She told those who would not use the "physicke" that they would never get well, and accordingly their diseases continued, contrary to the efforts of the physicians and surgeons.

4. She had the witches' marks.

In spite of her protestations of innocence, poor Margaret was hanged as a witch on Boston common in 1648. Those were the days when this law was on the statute books: "If any man or woman be a witch, that is, hath or consulteth with a familiar spirit, they shall be put to death."

Almost without exception in all the early settlements of Massachusetts and Connecticut the practice of medicine was carried on to a greater or less extent by the ministers. This union of medicine and religion was called by Cotton Mather "an angelic conjunction." The first medical treatise published in America was by one of these minister-physicians, Thomas Thatcher, and is entitled "Brief Rules for the Care of the Small Pocks," 1677.

Probably the most famous minister-physician was the Rev. Michael Wigglesworth, who was born in Yorkshire, England, October 28, 1631, and was brought to this country when a very young child. He entered Harvard College in 1647, and graduated with the class of 1651. After his graduation he was appointed a tutor at the college, and during that time prepared himself for the ministry and was ordained minister at Malden in August, 1656. After his ordination his health became so poor that he had to spend some time abroad, and was never of a very strong constitution. For nearly fifty years he served the church at Malden faithfully, both as minister and physician, as Cotton Mather said of him, "lively unto death." He died in 1705. Wigglesworth will always be remembered for his production of that sulphurous poem the "Day of Doom," which was very popular in its day, and also of a book called "Meat Out of the Eater." An extract from the "Day of Doom" may be interesting. The following is one describing the punishments of hell:

> "For day and night in their despite,
> Their torments smoak ascendeth;
> Their pain and grief have no relief,
> Their anguish never endeth;
> There must they ly and never dy
> Tho' dying every day;
> There must they dying ever ly
> And not consume away."

His death was lamented by Cotton Mather in the following poetical effort:

"His pen did once meat from the eater fetch,
And now he's gone beyond the eater's reach;
His body once so thin was next to none,
From hence he's to unbodied spirits flown;
Once his rare skill did all diseases heal,
And he does nothing now uneasy feel;
He to his paradise is joyful come
And waits with joy to see his Day of Doom."

His gravestone, bearing the following epitaph, still stands in the Malden burying-ground:

"Here lyes buried ye body of that faithful servant of Jesus Christ, ye reverand Mr. Michael Wigglesworth, pastour of ye Church of Christ at Maulden, who finished his work and entered upon an eternal sabbath of rest on ye Lord's Day, June ye 10, 1705, in ye 74 year of his age.

"Here lyes in silent grave below,
Maulden's physician of soul and body two."

Even in their dual capacity, these minister-physicians had a hard struggle to obtain a living, having to take their pay in anything they could get—cordwood, codfish, grain and such like produce. The records show that they were very poorly paid. A physician's fees were only six pence a visit at Hadley and Northampton in 1730, and eight pence at about Revolutionary times. Tooth-drawing cost eight pence extra. One independent New Haven doctor charged a shilling a visit, and got it.

A good idea of the physicians' fees in England a little earlier than this may be obtained from the funeral bill of John Dudley, who died in London in 1580. He was a brother of the Thomas Dudley who came to New England.

| | |
|---|---|
| To Dr. Astlow for his attendance during his sickness.......... | £6. |
| To the pottacarie for his bill ............................. | 5. |
| To Dr. Smith and Dr. Hector at the oapening of the bodie..... | 6. |
| To the surdgeons for oapening, searing, etc., of the bodie..... | 7. s5 |
| To a poore man that made an epitaphe ...................... | .s10 |

The total of his funeral expense was about three hundred and ten pounds. From this it will be seen that only about twenty-five pounds were for medical attendance, including the ten shillings that went to the "poore" man that made the epitaph.

The small fees of the olden time led the physicians to adopt all kinds of expedients to help them to get a living; most of them compounded their own medicines and sold them to their patients; this may account for some of the mammoth doses of those days. Others were innkeepers, schoolmasters and storekeepers; one was a butcher, and this in more ways than one.

A noted physician was Dr. Nathaniel Ames, of Dedham. He was doctor, innkeeper and also wrote almanacs. In 1725 he began the publication of his almanacs, and continued to do so for thirty-nine years. His house of intertainment was at the sign of the Sun, eleven miles from Boston.

Even the quack soon found his way to this new land. "Runnagate chyrugeons" and "physickemongers" they were called. In 1631 Nicholas Knapp was fined and whipped at Boston for pretending to cure the scurvy with a "water of noe worth" and which he sold "att a very deare rate." The same treatment might possibly be used with advantage on some of their nineteenth century descendants.

With the doctors making and selling their own medicines, and the people preparing many at home, there was no room left for the apothecary, and we find none at all until 1761, when Thomas Hanasyd Peck, of Boston, advertises "plain buttons, black thread, cards, lace and small bowstrings, verdegrees, copperas, men's and boys' felt hats, castor and logwood." I have found one advertisement of that time which may be that of an apothecary, in the *Boston Post-Boy* of Sept. 8, 1760:

JOHN LORING.

At his shop near the Great Trees. A fresh and General Assortment of Medicines, both Clinical and Galenical. Spices of all sorts; likewise Redwood, Logwood, Allum, Copperas, Brimstone, etc. N. B.—True Lockyer's Pills, Bateman's Drops, Stoughton and Duffey's Elixir, etc., etc.

Through this conglomeration of doctoring, preaching, butchering and tavern-keeping, a great deal of the science of medicine in this country has passed in its evolution down to the present time. Looking back at that little handful of resolute men who first obtained a foothold on the rock shores of New England, we can only be amazed at what has taken place since then. We should also be very thankful, too, for it was through the struggles and trials and efforts of our Puritan forefathers that many of the comforts and blessings we now enjoy have been produced. Some of them foresaw the future of this country in their day, and were glad. I can find no better closing than the prophetic words of Edward Johnson, in the *Wonder Working Providence of Zion's Saviour* in New England, 1654. "The Lord Christ intends to achieve greater matters by this little handful than the world is aware of."—*Medical Standard.*

